# Information-theoretic model averaging: a useful method for modelling exploratory secondary endpoints in clinical trials

**DOI:** 10.1186/1745-6215-14-S1-P111

**Published:** 2013-11-29

**Authors:** Daniel Lythgoe

**Affiliations:** 1 University of Liverpool, Liverpool, UK

## 

In clinical trials, highly exploratory secondary endpoints are common and the number of measured variables plausibly associated with response can be numerous, particularly if biomarkers are considered. Automated, single-best-model selection procedures are widely employed in such instances. Known issues are: 1) the precision of parameter estimates are estimated as if the selected model was prespecified and 2) useful competing models are disregarded. By contrast, information-theoretic approaches to model averaging (IT-MA) incorporate model selection uncertainty and combine parameter estimates across models.

Two IT-MA estimators exist: the ‘standard’ and the shrinkage estimator. The latter has been considered previously in the context of linear regression. We extend this by exploring IT-MA estimator properties in logistic regression simulation studies and compare results to minimum AIC selection (mAIC).

In the extreme case where no explanatory variables are related to response, the confidence interval coverage of both IT-MA estimators is near-nominal in sharp contrast to mAIC, even with a prudent number of events-per-variable. In the presence of collinearity we demonstrate that for r>0.7, both IT-MA estimators exhibit bias but have considerably lower variances than the full model. Interestingly, for true zero effects the standard IT-MA estimator is preferable whilst for non-zero effects the shrinkage estimator is preferable.

In conclusion, confidence interval coverage for IT-MA is superior to mAIC and both IT-MA estimators perform well even in the presence of collinearity. These results emphasise the usefulness of IT-MA as a tool for modelling exploratory secondary endpoints in clinical trials.

